# Novel Acylguanidine Derivatives Targeting Smoothened Induce Antiproliferative and Pro-Apoptotic Effects in Chronic Myeloid Leukemia Cells

**DOI:** 10.1371/journal.pone.0149919

**Published:** 2016-03-02

**Authors:** Alessandra Chiarenza, Fabrizio Manetti, Elena Petricci, Martial Ruat, Antonella Naldini, Maurizio Taddei, Fabio Carraro

**Affiliations:** 1 Department of Molecular and Developmental Medicine, University of Siena, Siena, Italy; 2 Department of Biotechnology Chemistry and Pharmacy, University of Siena, Siena, Italy; 3 CNRS, UMR-9197, Neuroscience Paris- Saclay Institute, Molecules Circuits Department, Signal Transduction and Developmental Neuropharmacology Team, Gif-sur-Yvette, France; 4 Istituto Toscano Tumori, Siena, Italy; Università degli Studi di Firenze, ITALY

## Abstract

The most relevant therapeutic approaches to treat CML rely on the administration of tyrosine kinase inhibitors (TKIs) like Imatinib, which are able to counteract the activity of Bcr-Abl protein increasing patient’s life expectancy and survival. Unfortunately, there are some issues TKIs are not able to address; first of all TKIs are not so effective in increasing survival of patients in blast crisis, second they are not able to eradicate leukemic stem cells (LSC) which represent the major cause of disease relapse, and third patients often develop resistance to TKIs due to mutations in the drug binding site. For all these reasons it’s of primary interest to find alternative strategies to treat CML. Literature shows that Hedgehog signaling pathway is involved in LSC maintenance, and pharmacological inhibition of Smoothened (SMO), one of the key molecules of the pathway, has been demonstrated to reduce Bcr-Abl positive bone marrow cells and LSC. Consequently, targeting SMO could be a promising way to develop a new treatment strategy for CML overcoming the limitations of current therapies. In our work we have tested some compounds able to inhibit SMO, and among them MRT92 appears to be a very potent SMO antagonist. We found that almost all our compounds were able to reduce Gli1 protein levels in K-562 and in KU-812 CML cell lines. Furthermore, they were also able to increase Gli1 and SMO RNA levels, and to reduce cell proliferation and induce apoptosis/autophagy in both the tested cell lines. Finally, we demonstrated that our compounds were able to modulate the expression of some miRNAs related to Hedgehog pathway such as miR-324-5p and miR-326. Being Hedgehog pathway deeply implicated in the mechanisms of CML we may conclude that it could be a good therapeutic target for CML and our compounds seem to be promising antagonists of such pathway.

## Introduction

Chronic myelogenous leukemia (CML) is a clonal myeloproliferative malignancy that arises in hematopoietic stem cells harboring the reciprocal translocation between chromosomes 9 and 22, thus resulting in the formation of the Philadelphia chromosome [1]. This translocation fuses the breakpoint cluster region (Bcr) and the Abelson kinase (Abl) genes, forming the Bcr-Abl oncogene that encodes the constitutively active cytoplasmatic tyrosine kinase (TK) Bcr-Abl [2,3], present in >90% of CML cases. The aberrant kinase activity of Bcr-Abl is responsible for CML initiation [4], and the consequent disease progresses through three phases (chronic proliferative phase, accelerated phase, and blast crisis phase), becoming more resistant to treatment in each successive phase. The last phase is also characterized by the presence of genomic instability and is ultimately fatal.

The finding that Bcr-Abl is the cause of the leukemic phenotype and that the TK activity of Abl is fundamental for Bcr-Abl-mediated transformation, make this kinase an important target for the development of specific therapies [5]. The advent of TK inhibitors (TKI) targeting Bcr-Abl has revolutionized the treatment of CML. Imatinib [6,7], which was the first Bcr-Abl inhibitor approved for CML therapy [8,9], has improved patients’life expectance and survival especially in the chronic phase. The occurrence of relapse, resistance [10–13], and the necessity of a continued chemotherapy led to the discovery of nilotinib [14,15], dasatinib [16], and bafetinib [17] that are much more active toward Bcr-Abl and are able to block imatinib-resistant CML, with the sole exception of the T315I Bcr-Abl mutation that is recognized by ponatinib [18], a third generation TKI. Dasatinib was approved by FDA in 2006 for adult patients (chronic phase CML) with resistance or intolerance to prior therapies, nilotinib was approved in 2010 for chronic phase CML patients, and ponatinib was approved in 2012 for T315I CML patients. At the end of 2012, also bosutinib, a dual Bcr-Abl/Src inhibitor, was approved by FDA for the treatment of adult patients with resistant CML in chronic, accelerated or blast phase [9]. Although such compounds demonstrated clinical efficacy in some cases of imatinib resistance, the problem of LSC insensitivity remained unsolved.

On the basis of these considerations, treatment of CML with currently available TKIs suffers from three major limitations. In fact, although Bcr-Abl expression is deeply reduced or abrogated in the majority of patients, the anti-CML drugs have not significantly improved survival in patients in blast crisis (BC) [19]. Moreover, imatinib is unable to kill leukemic stem cells (LSC) in CML [20,21] because LSC do not depend on Bcr-Abl activity for survival [22]. Finally, kinase domain mutations confer resistance to imatinib in several patients. Therefore, treatment of the blast crisis, eradication of LSC, and the insensitivity of resistant cells to imatinib still remain the major unsolved problems in the treatment of CML [19]. In this perspective, finding alternative strategies to overcome limitations of current therapies has acquiring growing importance.

Currently, several investigational approaches are under study in the attempt to prevent BC and to deplete LSC population. A potential approach for BC prevention is to interfere with the self-renewal properties of LSC [23]. In this context, a pivotal role for survival maintenance of LSC has been found for BCL6 [24], HIF1α [25], and Smoothened (SMO) [21,26,27]. Recent literature shows that Hedgehog (Hh) signaling pathway is clearly involved in expansion of Bcr/Abl-positive stem cells [27,28], and in functional regulation of CML in terms of self-renewal, proliferation, and apoptosis. Moreover, loss or pharmacological inhibition of SMO, an essential component of the Hh pathway, impairs LSC renewal, decreases the propagation of Bcr-Abl-driven CML, and reduces the growth of resistant CML [27].

Hh signaling pathway is highly conserved in vertebrates and it is involved in embryonic development, organogenesis and cell proliferation. In the absence of Hh ligands, Patched (Ptch) receptor inhibits the activation of the downstream protein SMO by keeping it blocked in an intracellular vescicle [29]. Upon binding of Hh ligands to Ptch receptor, such inhibition is released and the activation of SMO, followed by its migration on primary cilium cell membrane, leads to pathway activation which culminates in a signal transduction cascade. These events cause the nuclear translocation of the Gli family of transcription factors (Gli1-3), and the subsequent activation or inhibition of various cell cycle, proliferation and survival regulating genes such as the D-type cyclins, c-Myc and Bcl-2 [30–34]. As part of a feedback mechanism, Gli target genes also comprise members of the Hh pathway, such as Gli1 and Ptch1 [35,36]. Among the inhibitors of the Hh pathway that interact directly with SMO, vismodegib and sonidegib are the two compounds approved by FDA in 2012 and 2015, respectively, for the treatment of basal cell carcinoma (BCC). Other hedgehog inhibitor compounds have been studied for BCC, like CUR61414 [37], but though they showed a good activity both in vitro and on mice, they failed the clinical phase I studies [38].

On the other hand, preclinical studies are checking the possibility to induce apoptosis in BC cells by treatment with various drugs, alone or in combination. As an example, inhibition of Mek and farnesyl transferase [39], or treatment with a dual Bcr-Abl/Jak2 inhibitor [40], as well as p53 stabilization [18] induce apoptosis and death in human BC CML K-562 cells. Importantly, this cell line expresses all the Hh signaling molecules, including sonic Hh (Shh), Ptch, SMO and Gli1 [41].

Taken together, these results suggest that small molecules able to inactivate the Hh pathway by blocking SMO could be in principle useful either to inhibit BC cell proliferation or, at the same time, to deplete population of LSC whose survival, self-renewal, and expansion is strongly dependent on the Hh pathway [27]. Moreover, a combination therapy comprised of the currently available Bcr-Abl inhibitors and small molecules able to block SMO could represent a very promising and effective tool to deplete CML cells, overcome chemotherapy resistance, eradicate LSC, and thus potentially cure CML.

MicroRNAs (miRNAs) are a class of small non-coding cellular RNAs that are responsible for messenger RNA translational inhibition or degradation. In several human cancers, a downregulated miRNA signature with high Hh signaling does exist. Downregulation of these miRNAs allows high levels of expression of Hh-dependent genes leading to tumour cell proliferation. As an example, miR-324-5p was shown to target the activator components of the Hh pathway, SMO and Gli1, thereby suppressing progenitor and tumour cell growth [42]. Moreover, it has been demonstrated that upregulation of SMO is associated with reduced expression of miRNA-326 [43].

Within a drug design project aimed at identifying new small molecules acting as Hh inhibitors, we have recently found a class of compounds with a very impressive ability to inhibit SMO by direct interaction [44,45]. In particular, in previous studies, compound MRT-83, belonging to the chemical class of acylguanidines substituted with a phenyl group, appeared to be one of the most potent SMO antagonists known so far [46]. On this basis, we planned to design new MRT-83 analogues and to check their ability to block growth and proliferation of two human BC CML cell lines (namely, K-562 and KU-812) and among the tested compounds we found that the one which possessed the phenylethyl terminal group (MRT92) was particularly active toward Hh pathway.

## Results and Discussion

### Specificity for Hh pathway

**Fig 1:**
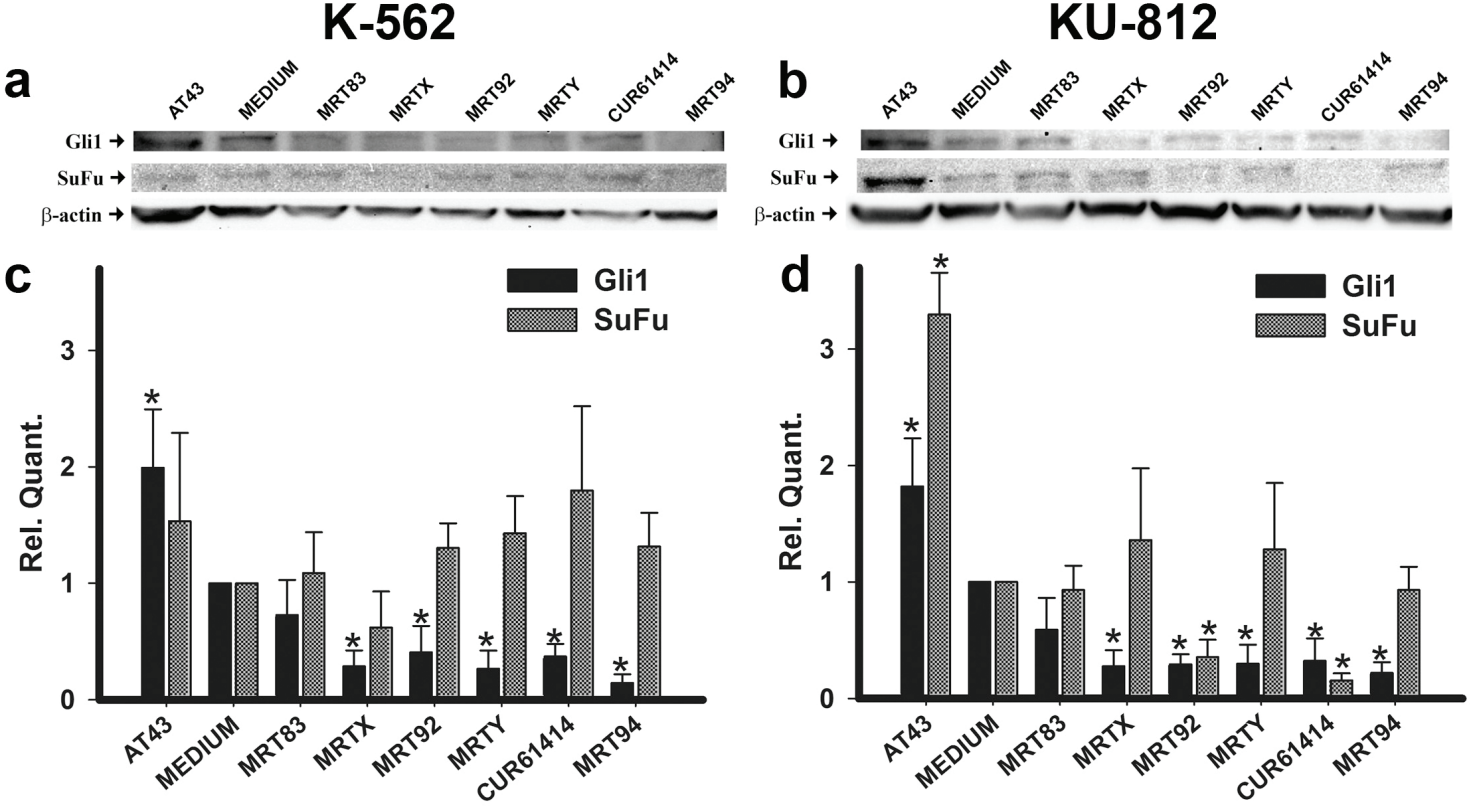
Effects of compounds and controls on Gli1 and SuFu protein expression. Effects of compounds MRT83, MRTX, MRT92, MRTY, MRT94 and control compounds after a 24 h treatment at 20 μM on Gli1 and SuFu protein expression in K-562 cells (a) and KU-812 cells (b) and quantification relative to medium expression (c-d). β-actin was used as loading control. Data are representative images, quantifications are expressed as the means ± SEM of three independent experiments. *p<0.05 vs medium.

**Fig 2:**
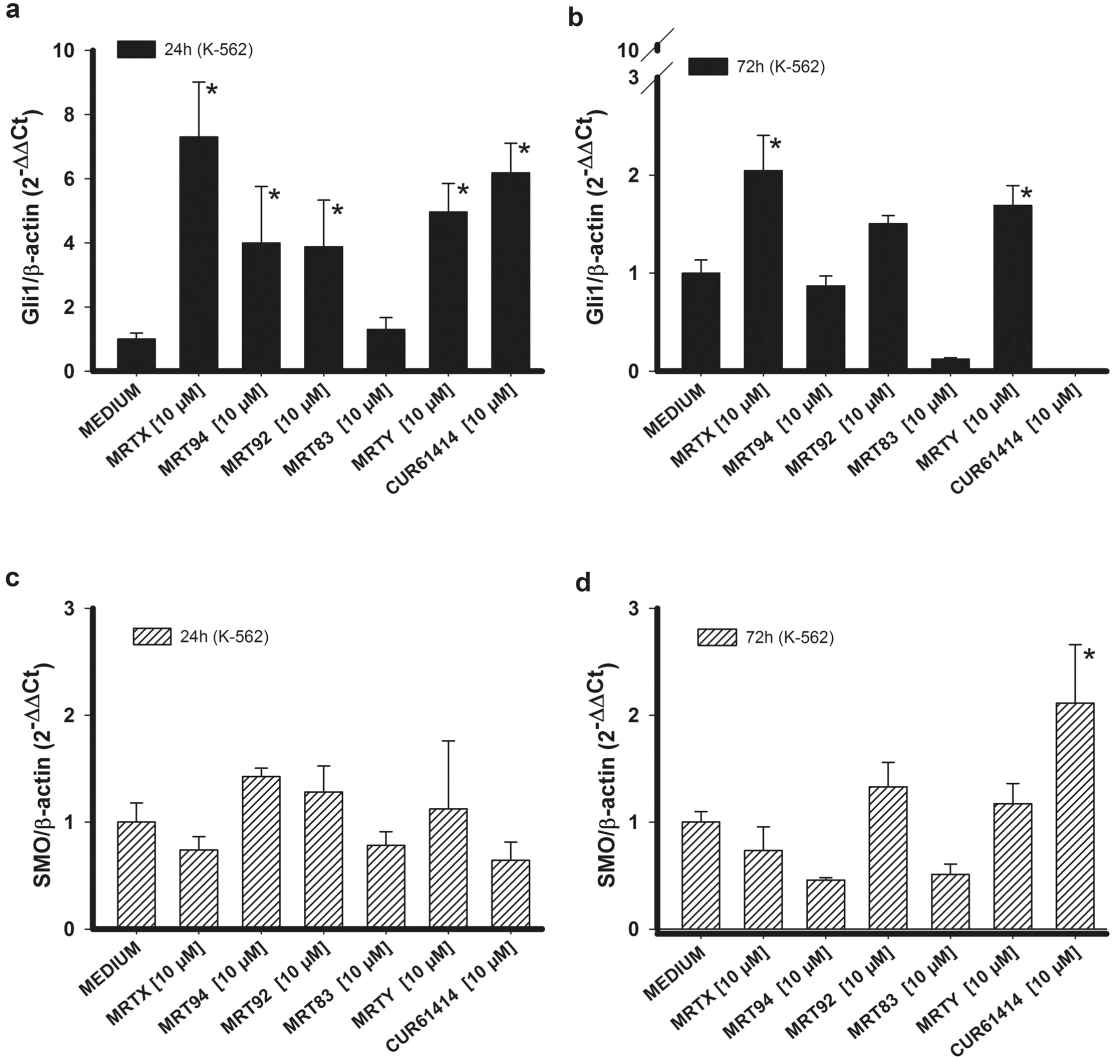
Gli1 and Smo RNA expression in K-562 cells. Effects of compounds MRTX, MRT94, MRT92, MRT83, MRTY and control compound in K-562 cells on Gli1 RNA expression after a 24h (a) or 72h (b) treatment and on SMO RNA expression after a 24h (c) or 72h (d) treatment at 10 μM. Data are expressed as the means ± SEM of four independent experiments performed in triplicate. *p<0.05 vs medium.

Western blotting analysis was applied to evaluate the ability of the compounds to affect Gli1 and suppressor of fused (SuFu) protein expression. Compounds effects were compared with the activity of CUR61414 used as reference compound and AT43 (a known SMO agonist) [37]. As shown in Fig 1a and quantified in Fig 1c treatment of K-562 and KU-812 (Fig 1b and 1d) cells with the compounds MRTX, MRT94, MRT92, MRT83, at 20 μM for 24 h, significantly reduced Gli1 protein concentration in comparison with non-treated control. A similar decrease of Gli1 protein was found by treatment with CUR61414. We were not able to determine any significant effect of MRTY on both cell lines. While on the contrary, as expected, AT43 was able to significantly increase Gli1 expression. These results showed a negative modulation of Hh pathway by our SMO inhibitors through the reduction of Gli1 protein expression. We also investigated the effect of our compounds on SuFu which is known to be a regulator of Gli proteins counteracting Spop activity, thus preventing Gli factors degradation [47]. On K-562 cells our compounds were not able to induce any significant modification of SuFu and even AT43, despite inducing a significant increase of Gli1, did not produce an increase in SuFu. On the contrary, on KU-812 cells the compounds MRT92 and CUR61414 were able to significantly reduce the amount of expressed protein, while AT43 induced a significant increase that correlated with Gli1 increase. The reduction of SuFu, even if not always significant, is a further confirmation of the ability of tested compounds to interact with Hh pathway. Given the specificity of these compounds to target Hh pathway, their effects on RNA expression were evaluated for the most important pathway components (i.e., Gli1 and SMO). As expected, no significant changes of the Gli1 and SMO RNA expression levels were found in both cell lines after a 3 h incubation with the studied compounds (data not shown). On the contrary, Gli1 RNA expression was increased in K-562 cells by a 24 h treatment with 10 μM inhibitor (Fig 2a), with the sole exception of MRT83. This enhanced expression could be considered as a compensatory effect that balances Gli1 protein level reduction consequent to pathway blockade. Only MRTX and MRTY showed a residual effect after 72 h (Fig 2b). SMO RNA levels were unchanged by treatment of K-562 cells for 24 (Fig 2c) and 72 h (Fig 2d), probably because the compounds act only by binding the protein and preventing its ciliar translocation, without effectively reducing the cellular amount of SMO. In this way, no compensatory mechanisms are required to be activated by the cell.

**Fig 3:**
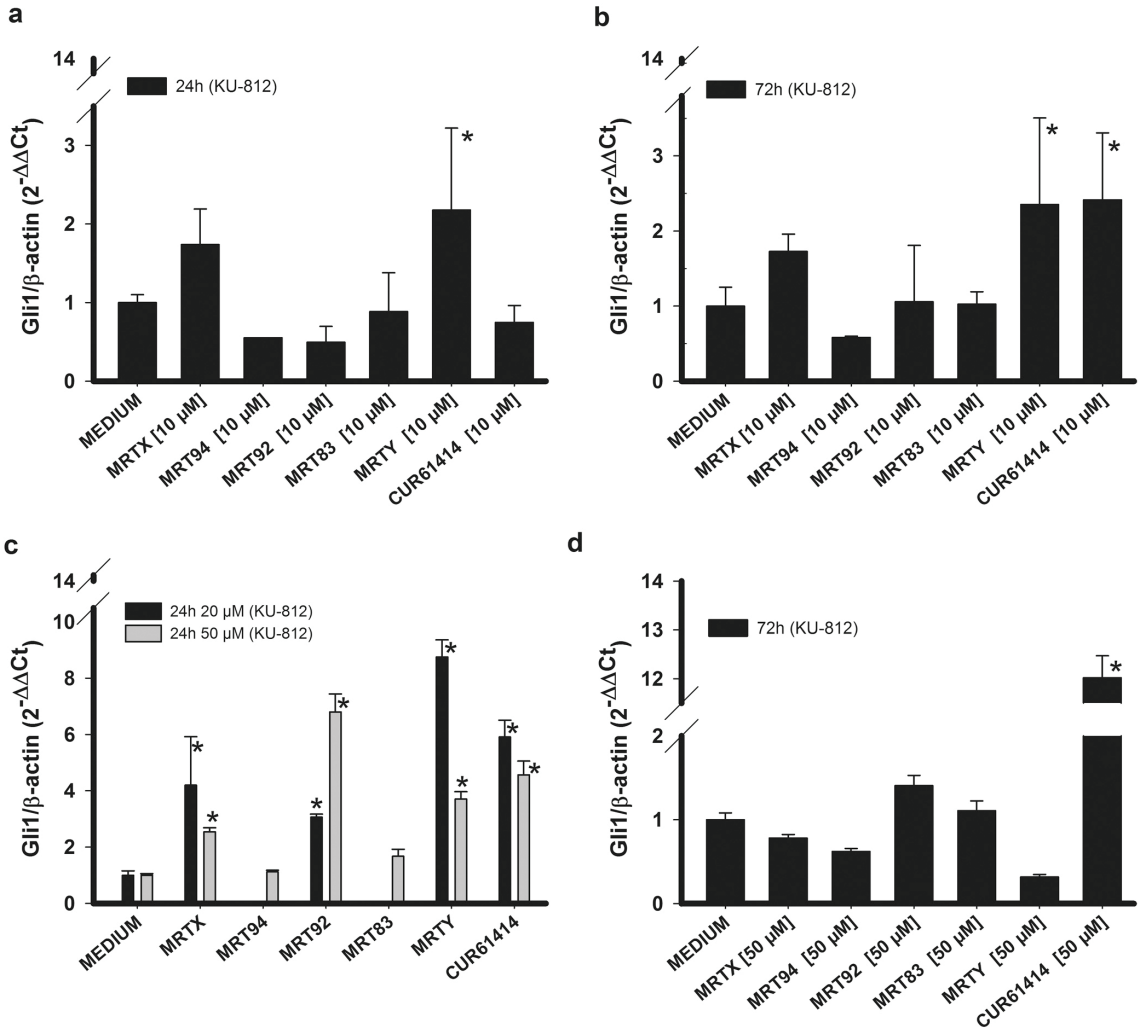
Gli1 RNA expression in KU-812 cells. Effects of compounds MRTX, MRT94, MRT92, MRT83, MRTY and control compound in KU-812 cells on Gli1 RNA expression after 24h (a) or 72h (b) treatment at 10 μM and treatment at 20 and 50 μM after 24h (c) or 72h (d). Data are expressed as the means ± SEM of three independent experiments performed in triplicate. *p<0.05 vs medium.

On the other hand, KU-812 cells responded to the studied compounds in a different way. In fact, only 10 μM MRTY induced an increase of Gli1 RNA expression after 24 h treatment (Fig 3a) and its effect was maintained at 72 h (Fig 3b). Increasing compound concentrations to 20 and 50 μM led MRTX and MRT92 (in addition to MRTY) to produce an effect after 24 h (Fig 3c), not maintained at 72 h (Fig 3d). Compounds MRT94 and MRT83 were tested only at 50 μM since they did not show any significant activity even at this concentration. These results suggest that KU-812 cells are less sensitive to the studied compounds in comparison to K-562 cells. Consequently, higher compound concentrations were required to determine a biological effect.

**Fig 4:**
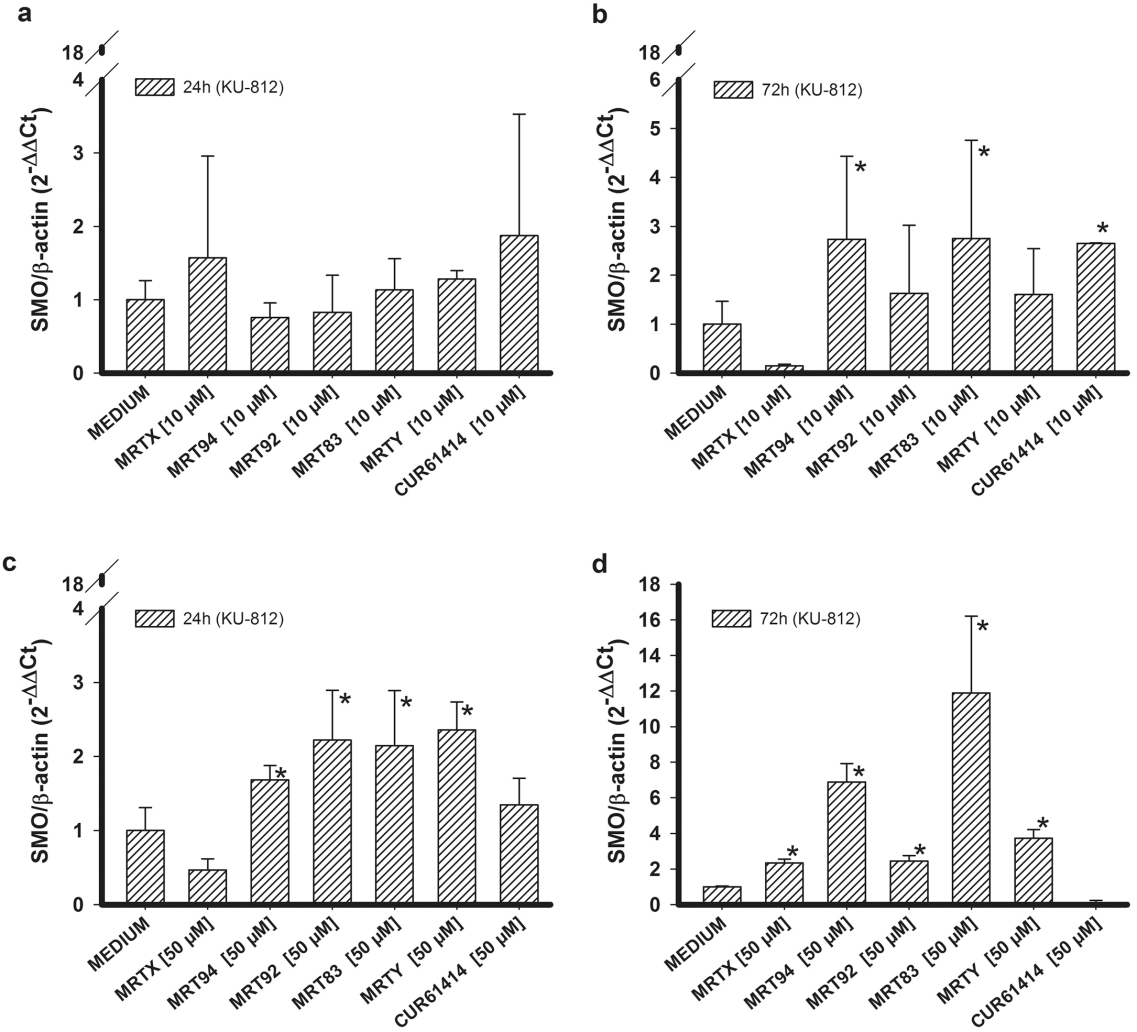
SMO RNA expression in KU-812 cells. Effects of compounds MRTX, MRT94, MRT92, MRT83, MRTY and control compound in KU-812 cells on SMO RNA expression after 24h (a) or 72h (b) treatment at 10 μM and treatment after 24h (c) or 72h (d) at 50 μM. Data are expressed as the means ± SEM of three independent experiments performed in triplicate. *p<0.05 vs medium.

A compound concentration of 10 μM did not affect SMO RNA expression after a 24 h treatment (Fig 4a), while increased SMO RNA levels were found after a 72 h treatment with MRT94 and MRT83 (Fig 4b). Increased concentrations (50 μM) of MRT94-Y led to higher SMO RNA expression levels after 24 h (Fig 4c), while all tested compounds were able to increase SMO RNA expression after a 72 h treatment (Fig 4d). In summary, differently from what found in K-562 cells, an increase of SMO RNA expression was found in KU-812 cells after a 10 μM treatment for 72 h, and after a 50 μM treatment after 24 and 72 h. These results suggest that a 10 μM concentration is too low to induce a rapid effect (within 24 h), while higher concentrations (50 μM) exhibit their effects already after 24 h and maintain them for 72 h at least.

### Effects on miRNAs expression

**Fig 5:**
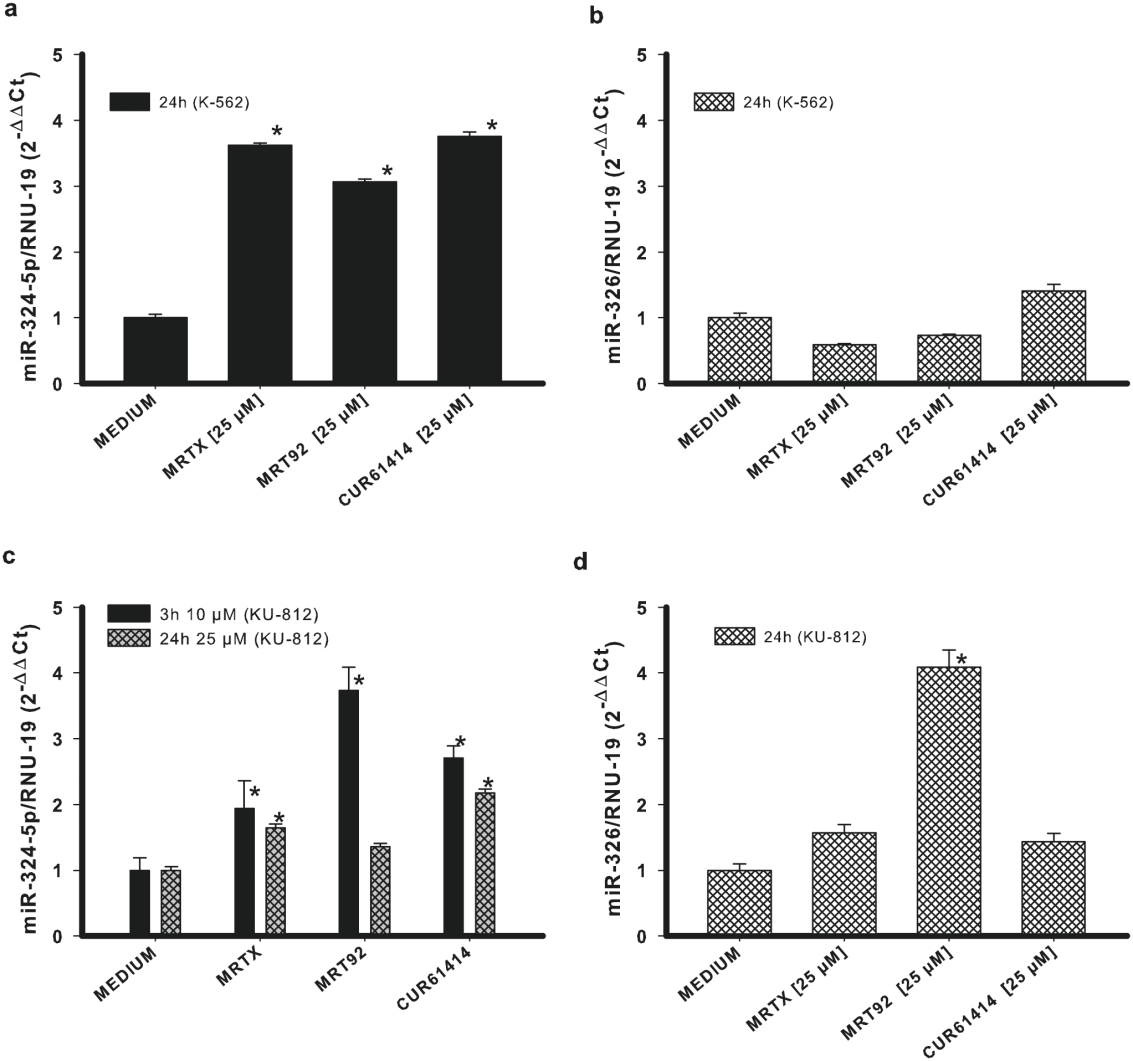
Changes in miRNAs expression. Effects of compounds MRTX, MRT92 and control compound in K-562 cells (a-b) or KU-812 cells (c-d) on miRNA324-5p and miRNA-326 expression. Data are expressed as the means ± SEM of three independent experiments performed in triplicate. *p<0.05 vs medium.

To confirm the specificity of our compounds towards Hh pathway, we investigated the effects they exerted on two miRNAs closely related to the Hedgehog pathway: miRNA-324-5p and miRNA-326. They both suppress the pathway activator SMO, and miR-324-5p also regulates the downstream transcription factor Gli1[42]. For this analysis, the two compounds with the best activity for both cell lines have been selected. Both K-562 and KU-812 cells were treated with 25 μM MRTX and MRT92 for 24 h. Results show a significant increase of miR-324-5p in both cell lines and the greatest effect toward KU-812 cells was found after 3 h of treatment (Fig 5a and 5c). In addition, MRT92 at 25 μM after 24h of treatment was able to increase miR-324-5p but did not reach significativity. These results are in agreement with the hypothesis that compounds negatively regulate SMO and inactivate the Hh pathway by the reduction of Gli1, thus inducing an increase of miRNA324-5p level. According to previous results showing that SMO expression was not affected in K-562 cells, miR-326 expression did not change in the same cell line (Fig 5b). On the contrary, a significant increase of its expression was found in MRT92-treated KU-812 cells (Fig 5d).

It is noteworthy that changes of the miRNAs levels are in agreement with Gli1 and SMO RNA variations in both tested cell lines. In fact, the levels of both miRNA-324-5p and its target (Gli1) increase after a 24 h treatment in the tested cell lines; the amounts of miRNA-326 and its target (SMO) remain both unaltered in K-562 cells and increase in KU-812 cells with the only exception of miRNA-326 in the sample treated with compound MRTX. This parallel level of expression of the miRNAs and the respective target genes is due to the activation of a control mechanism which promotes miRNAs increase to prevent the uncontrolled production of the target gene RNA.

### Antiproliferative activity

**Fig 6:**
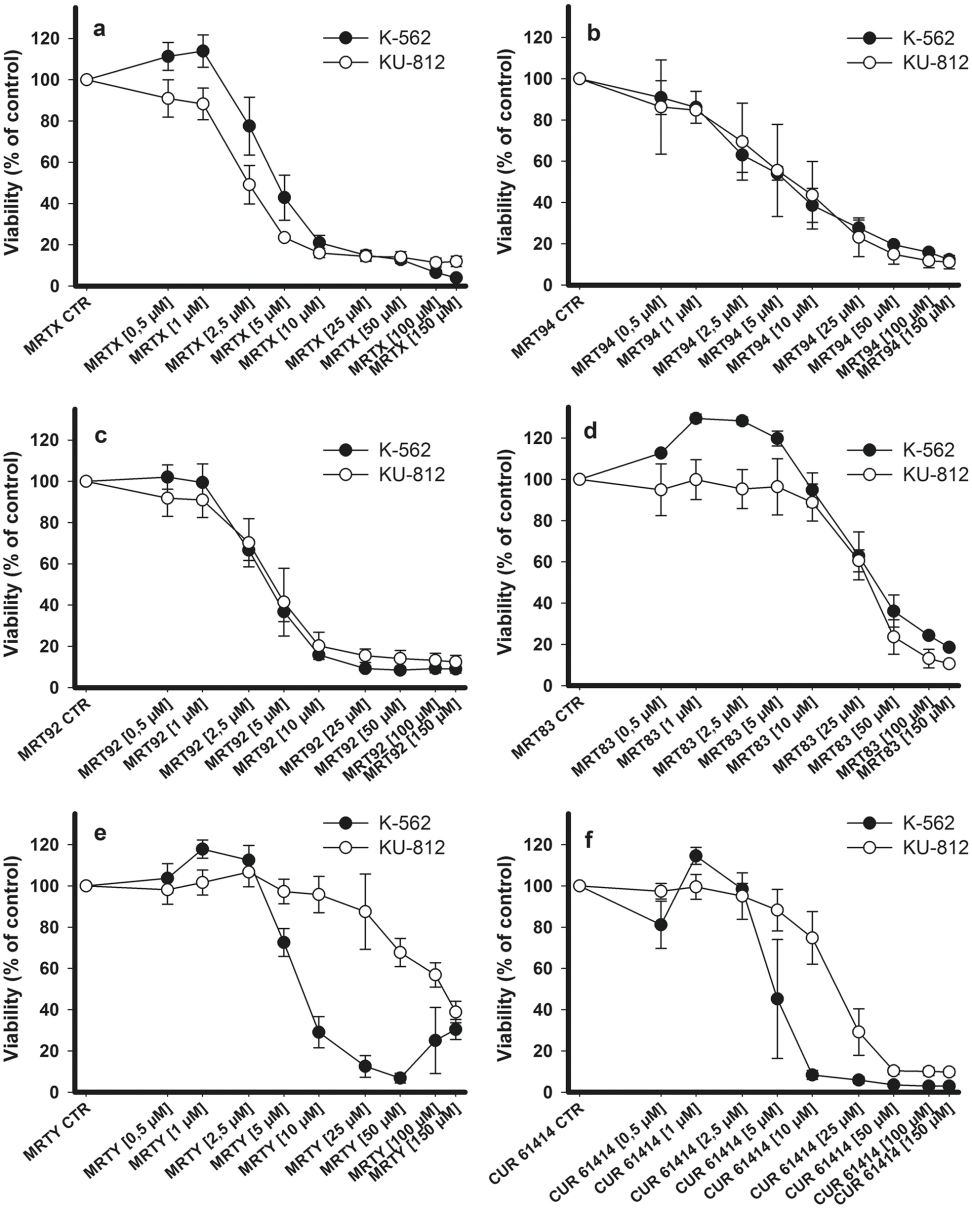
Effects of compounds in K-562 cells and KU-812 cells viability. Effects of compounds MRTX (a), MRT94 (b), MRT92 (c) MRT83 (d), MRTY (e) and CUR61414 (f) (0.5–150 μM) in K-562 cells (black dots) and KU-812 cells (white dots) viability Values are the mean ± SEM for three independent experiments performed in triplicate.

Ability of the new compounds and CUR61414 to affect viability of K-562 and KU-812 cell lines (Fig 6) was checked by resazurin proliferation assay. The IC_50_ values obtained for each compound are listed in Table 1. A concentration-dependent antiproliferative activity of compounds comparable to or better than that of CUR61414 was found toward both cell lines. In particular, MRT92 and MRTY showed IC_50_ values lower than 10 μM in K-562 cells (7.7 and 7.8 μM, respectively, versus 14 μM found for CUR61414). IC_50_ values for MRTX and MRT92 toward KU-812 cells were respectively 5.5 and 7.2 μM (27 μM for CUR61414). The remaining compounds showed a two-digit micro molar IC_50_.

**Table 1:** Antiproliferative effects of the new compounds toward K-562 and KU-812 cell lines.

Compounds	IC_50_ (μM)	
	K-562 CELL LINE	KU-812 CELL LINE
MRTX	26.49 ± 2.4	5.48 ± 1.65
MRT94	22.99 ± 4.8	20.02 ± 14.96
MRT92	7.67 ± 2.62	7.22 ± 1.52
MRT83	58.64 ± 15.19	55.07 ± 9.25
MRTY	7.82 ± 4.79	>100
CUR61414	14.41 ± 5.91	27.11 ± 12.94

### Pro-apoptotic activity and autophagy

**Fig 7:**
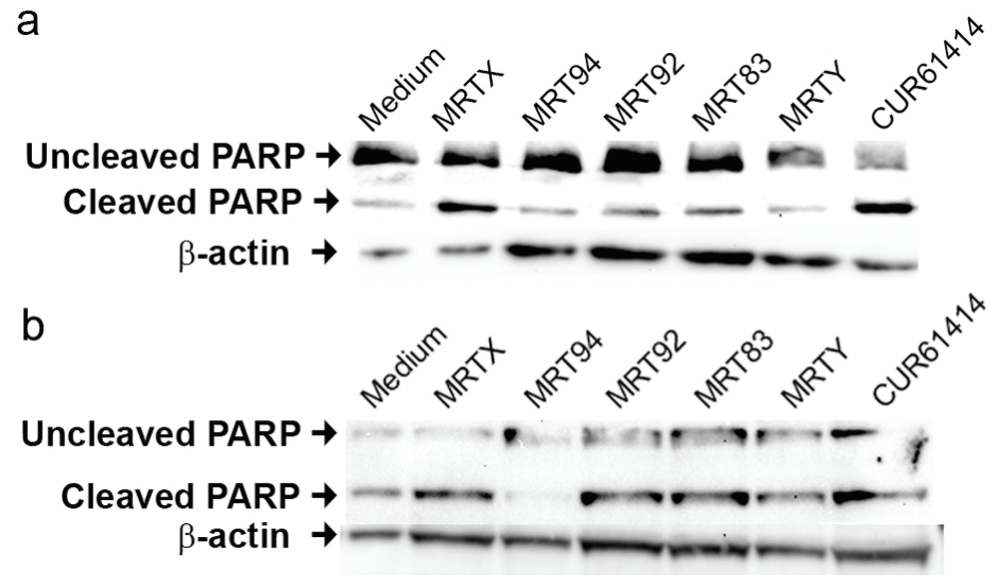
Pro-apoptotic activity of the compounds on PARP cleavage. Effects of compounds MRTX, MRT94, MRT92, MRT83, MRTY and control compound after a 72h treatment at 10 μM on PARP cleavage in K-562 cells (a) and KU-812 cells (b). β-actin was used as loading control. Data are representative images of three independent experiments.

Prompted by their ability to reduce CML cell line proliferation, the new compounds were also checked for pro-apoptotic activity versus poly-ADP-ribose-polymerase (PARP). We used a single representative compound concentration of 10 μM. Immunoblot analysis of uncleaved and cleaved PARP indicated that a significant PARP cleavage did not occur in K-562 cells after 72 h of treatment except for MRTX treated cells (Fig 7a), while tested compounds, with the exception of MRT94, led to an enhancement of the cleaved PARP in KU-812-cells that were potently induced to apoptosis after 72 h of treatment (Fig 7b).

**Fig 8:**
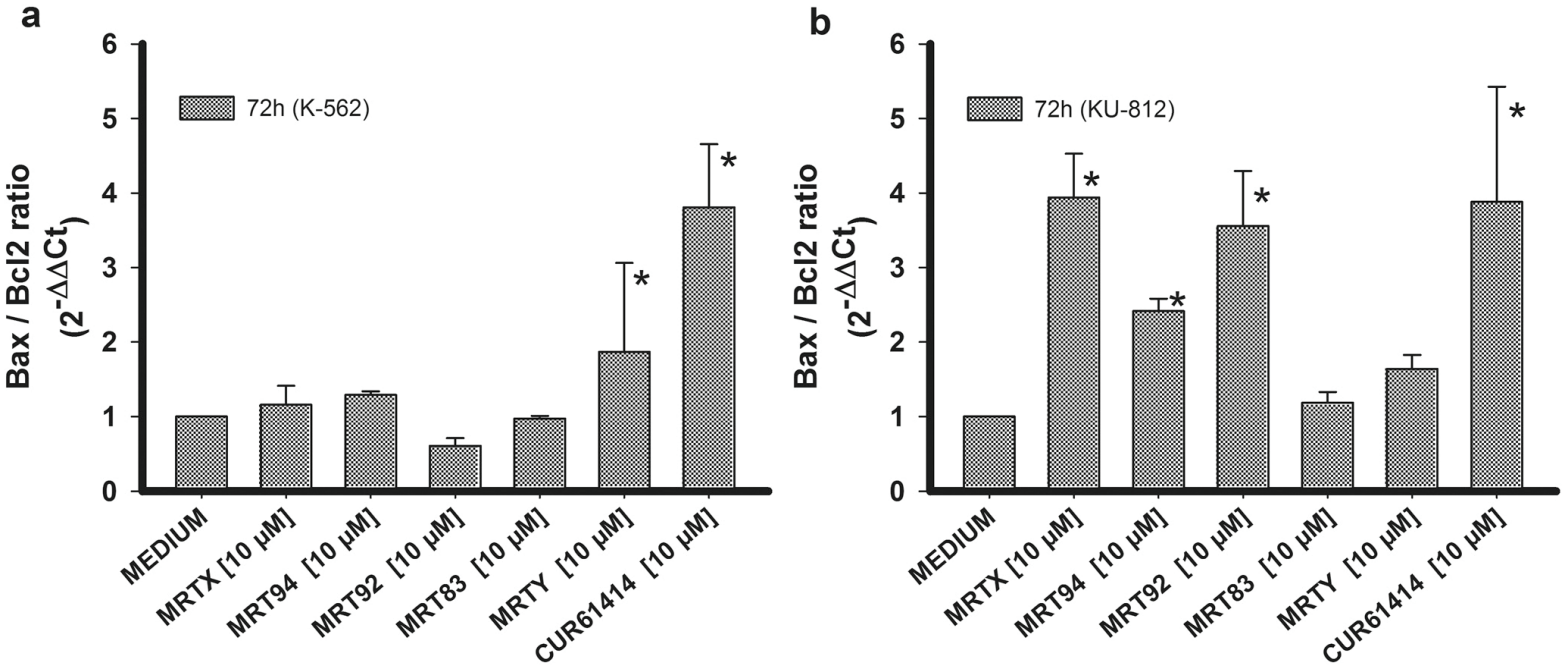
Pro-apoptotic activity of the compounds expressed as ratio between Bax and Bcl-2 RNA levels. Effects of compounds MRTX, MRT94, MRT92, MRT83, MRTY and control compound after a 72h treatment at 10 μM on Bax/Bcl2 RNA ratio in K-562 cells (a) and KU-812 cells (b). Data are expressed as the means ± SEM of three independent experiments performed in triplicate. *p<0.05 vs medium.

Moreover, the expression of Bax and Bcl-2 RNA levels was also investigated. In fact, the ratio between Bax and Bcl-2 RNA expression is a critical determinant to induce cells toward apoptosis and represents a direct index of the induction of the apoptotic process [48], thus helping in exploring the apoptosis induction. In agreement with results of the PARP assay, no significant pro-apoptotic effect was measured in K-562 cells after 72h of treatment except for samples treated with MRTY (Fig 8a), while MRTX, MRT94, and MRT92 were able to induce apoptosis in KU-812 cells after 72h (Fig 8b).

**Fig 9:**
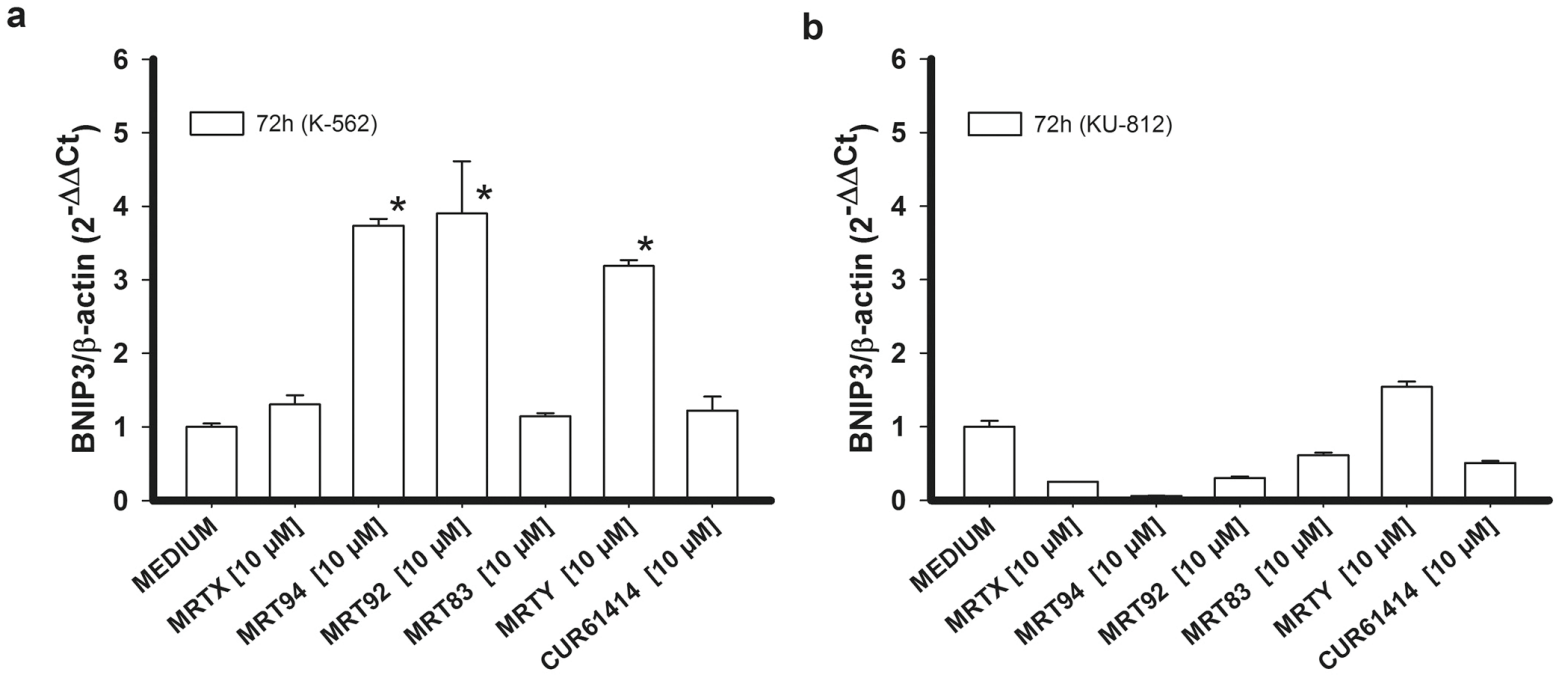
Pro-autophagic activity of the compounds expressed as BNIP3 RNA levels. Effects of compounds MRTX, MRT94, MRT92, MRT83, MRTY and control compound after a 72h treatment at 10 μM on BNIP3 RNA ratio in K-562 cells (a) and KU-812 cells (b). Data are expressed as the means ± SEM of three independent experiments performed in triplicate. *p<0.05 vs medium.

We further evaluated the expression of BNIP3, a protein that has been shown to be correlated to autophagy [49]. Seventy-two hours exposure to compounds lead to an increase in BNIP3 expression, particularly this was significant in samples treated with MRT94, MRT92, and MRTY in K-562 cells (Fig 9a). This result is in agreement with previous findings [49] that show an induction of autophagy in Bcr-Abl-positive CML cells by inhibition of Hh pathway. Differently from K-562 cells, BNIP3 expression in KU-812 cells was not significantly increased (Fig 9b), on the contrary the level of BNIP3 was reduced by the same compounds that elicited apoptosis.

Even if it is reported in literature [50] that the Hh pathway blockade produce an apoptotic response in K-562 cell line, this is not accordant with our results. But since we know that an increase of BNIP3 indicate an early cell damage which most likely will lead to an apoptotic response in longer time frames the differences are probably only due to experimental detection timing.

The reported results demonstrated that some of the tested compounds were able to induce autophagy in K-562 cell line mediated, as shown, by an increase of BNIP3 RNA levels. MRTX and MRT92 induced apoptosis on KU-812 cells as shown by the increase of cleaved PARP and the ratio of Bax/Bcl2. MRT94 was able to significantly increase the ratio of Bax/Bcl2 but did not show any increase in PARP cleavage; probably this compound may require a longer time to fully activate the caspase cascade.

### Proliferation comparison on K-562 cells

**Fig 10:**
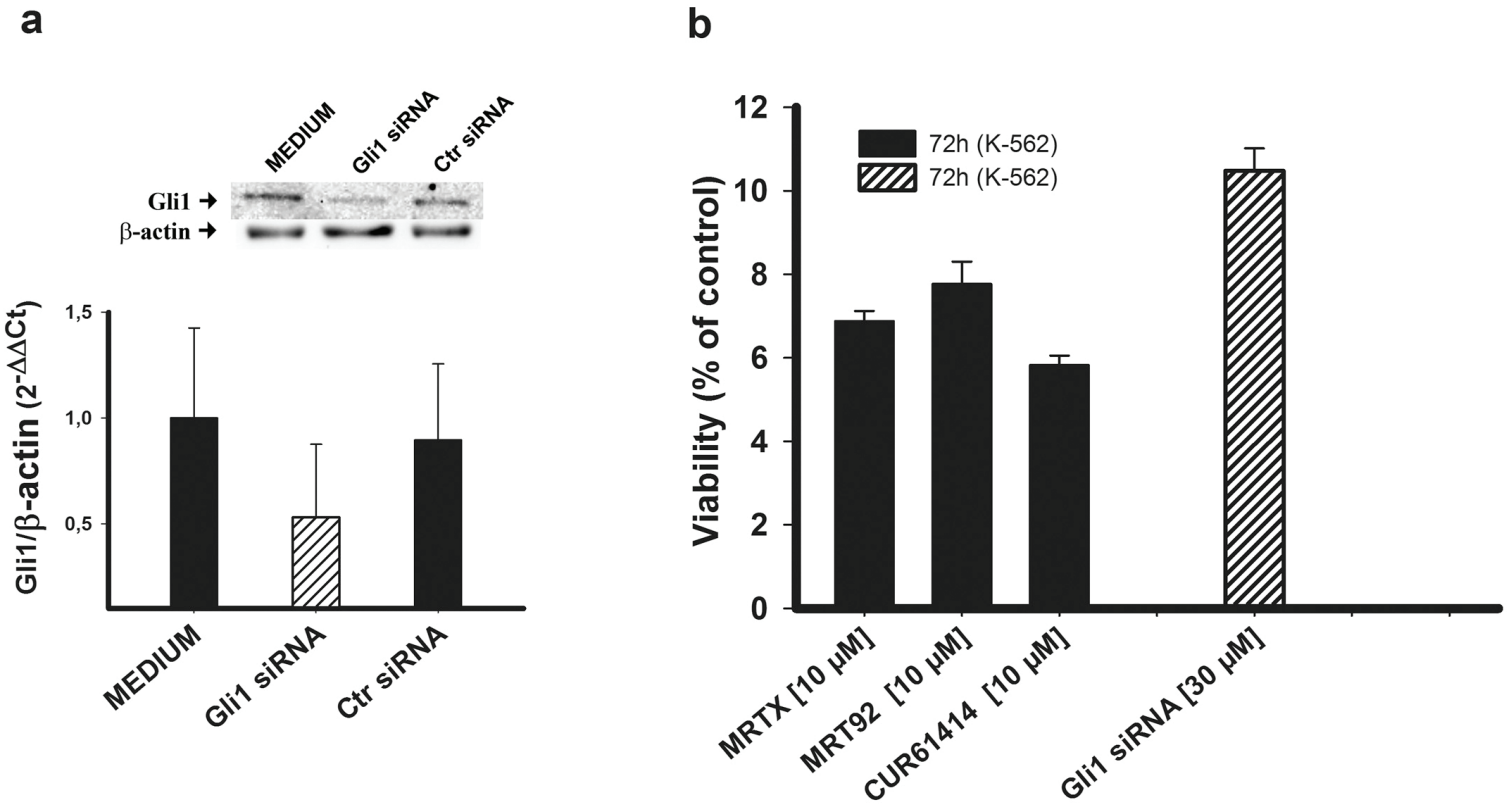
Effects Gli1 siRNA and compounds in K-562 cells viability. Effect of Gli1 siRNA and Ctr siRNA on Gli1 protein and RNA expression (a). Percent viability of non silenced K-562 cells treated with MRTX and MRT92 and Gli1 silenced K-562 cells (b). Data are expressed as the means ± SEM of three independent experiments performed in triplicate.

We evaluated the inhibition of proliferation on K-562 cells after either Gli1 gene silencing or treatment with compounds that have proven to be able to reduce Gli1. For this experiment, we chose MRTX and MRT92. Gli1 siRNA was inserted by electroporation in K-562 cells and this elicited a reduction on Gli1 expression (Fig 10a). Blocking Hh pathway by Gli1 gene silencing led to a significant reduction on cells viability that was comparable with the reduction of viability in non-silenced K-562 cells induced by MRTX and MRT92 (Fig 10b). In both cases inhibition of cell proliferation was about 90%. It is of particular interest that pathway blockade with our compounds showed the same inhibition of proliferation of biological pathway blockade through Gli1 gene silencing.

The current treatment for CML is based on TKIs [6,7,11]. Despite their efficacy, TKIs present several limitations as their inability to improve survival in patients in BC [19], the development of resistance [19] and their inability to kill LSC which represent the reservoir of the disease and the major cause of relapse [20,21].

Given the relationship between Hh-SMO pathway activation and CML progression from LSC to BC, combined with the ability of our compounds either to block SMO or to inhibit CML cell growth and proliferation, our compounds seem to be particularly suitable for a promising therapeutic approach toward CML. A combination therapy comprised of the currently available Bcr-Abl inhibitors (such as ponatinib that is also able to target imatinib-resistant cells) and new small molecules that are able to block SMO could represent a very promising and effective tool to deplete CML cells also in blast crisis, overcome chemotherapy resistance, and eradicate LSC, as already reported in some literature for vismodegib and ponatinib [51].

Among our compounds the one with phenylethyl terminal group (MRT92) appears to be very specific towards Hh pathway as it strongly decreases Gli1 protein expression and modulates Gli1 and SMO RNA levels and miRNAs in both tested cell lines. Furthermore it demonstrated an impressive ability to inhibit proliferation in both tested cell lines with IC_50_ values far below 10 μM, it induced apoptosis in KU-812 and seems to provoke autophagy in K-562 cell line. In conclusion our study has proven that MRT92 is certainly a promising therapeutic compound, and the best candidate for further experimental investigations.

## Experimental Section

Synthesis of SMO antagonists is described in S1 Appendix and illustrated in S1 Fig of Supporting Information.

### Cell lines and treatments

Human CML K-562 cells (American Type Culture Collection) in blast crisis and human CML KU-812 cell line (American Type Culture Collection) in myeloid blast crisis, both expressing Hh signaling pathway and carrying Philadelphia chromosome, were employed for biological assays. Cell lines were cultured in RPMI 1640 medium (Euroclone, Devon, UK), supplemented with 10% or 20% FCS, respectively, 1% L-glutamine 2 mM, streptomycin 100 μg/ml, and penicillin 100 U/mL (Euroclone, Devon, UK), and were maintained in a humidified atmosphere at 37°C and 5% CO_2_.

When indicated, K-562 and KU-812 cells were treated with compounds (MRTX, MRT94, 2, MRT83, MRTY), with the reference compound CUR61414, or with an agonist of the Hh pathway (AT43) for 3, 24 or 72 h at indicated compound concentration.

### Proliferation assay

Cell proliferation was evaluated by resazurin fluorescent method. Cells were starved overnight with RPMI 1640 culture medium supplemented with 0.5% FCS, 1% L-glutamine, and antibiotics (100 μg/mL streptomycin and 100 U/ml penicillin), and maintained in a humidified atmosphere at 37°C and 5% CO_2_.

Later, the medium was removed and the culture was refreshed with new medium at the usual concentration of FCS. Cells were plated at a concentration of 10^5^ cells/well in a 96 multiwell plate. Then, scalar concentrations of each compound ranging from 0.5 μM to 150 μM or a fixed concentration of compound (10 μM), or no compounds were added to the cells and the plate was incubated in a humidified atmosphere at 37°C and 5% CO_2_ for 72 h. Six hours before the end of incubation, resazurin was added at a final concentration of 320 μM and fluorescence was evaluated by fluorimetric analysis employing FLUOstar OPTIMA plate reader (BMG LABTECH, Offenburg, Germany) at an excitation wavelength of 530 nm and emission wavelength of 590 nm.

### RNA isolation and quantitative real time PCR

To determine Gli1 and SMO expression, K-562 and KU-812 cells were starved overnight with RPMI 1640 culture medium supplemented with 0.5% FCS, 1% L-glutamine, and antibiotics (100 μg/mL streptomycin and 100U/mL penicillin), and maintained in a humidified atmosphere at 37°C and 5% CO_2_. Later, the medium was removed and the culture was refreshed with new medium at the usual concentration of FCS. Cells were plated at a concentration of 3.5 x 10^5^ cells/ml and added with each compound (10 or 50 μM) for 24 or 72 h.

Total RNA isolation was performed by cell lysis with TRI-Reagent (Ambion, Foster City, USA) by taking the upper aqueous phase obtained after centrifugation at 1000g for 10 min. RNA was then washed in isopropanol and cool 75% ethanol, resuspended in nuclease-free water and kept at -20°C for further analysis.

MicroRNA isolation was performed by miRCURY^™^ RNA Isolation Kit—cell & plant (EXIQON, Vedbaek, Denmark) according to manufacturer’s instruction.

cDNA from total RNA extracted in TRI-Reagent was then synthesized using the iScript^™^ cDNA Sinthesis Kit (Bio-Rad Laboratories, Hercules, USA) and qRT-PCR analysis of Bax, Bcl-2, BNIP3, SMO and Gli1 RNA expression was performed on cDNAs by using iQ^™^ SYBR Green Supermix (Bio-Rad Laboratories, Hercules, USA).

Primer were designed using Primer3 [52,53] and purchased from Invitrogen (Carlsbad, USA), sequences are reported in Table 2. Data were analyzed with iQ^™^ 5 Optical System Software, Security Edition (Bio-Rad Laboratories, Hercules, USA). All values were normalized to β-actin endogenous control and RNA relative expression was measured using the 2^-ΔΔCt^ method.

**Table 2:** Primer sequences.

Gene name	Forward primer sequence	Reverse primer sequence
BAX	AGAGGATGATTGCCGCCGT	CAACCACCCTGGTCTTGGATC
Bcl2	TCCATGTCTTTGGACAACCA	CTCCACCAGTGTTCCCATCT
BNIP3	ACCCTCAGCATGAGGAACAC	TTCATCAAAAGGTGCTGGTG
Gli1	ACCCCCTGGACTCTCTTGAT	GGAATTCTGTTTCCCCAGGT
SMO	GGGAGGCTACTTCCTCATCC	GGCAGCTGAAGGTAATGAGC

cDNA from RNA isolated by miRCURY^™^ RNA Isolation Kit—cell & plant was synthesized using miRCURY^™^ LNA Universal RT microRNA PCR (EXIQON, Vedbaek, Denmark) according to manufacturer’s instruction and qRT-PCR analysis of Gli1, SMO, miR-324-5p, miR-326 RNA expression was performed on cDNAs by using ExiLENT SYBR^®^ Green master mix (EXIQON, Vedbaek, Denmark) and MicroRNA LNA^™^ PCR primers. Data were analyzed with iQ^™^ 5 Optical System Software, Security Edition. All values were normalized to non-coding RNA U6, and RNA spike-ins were used as controls for isolation, cDNA synthesis and PCR. RNA relative expression was measured using the 2^-ΔΔCt^ method.

### Protein expression

To assess Gli1, SuFu and ß-actin protein production and PARP cleavage, K-562 and KU-812 cells were starved overnight with RPMI 1640 culture medium supplemented with 0.5% FCS, 1% L-glutamine and antibiotics (100 μg/ml streptomycin and 100 U/ml penicillin), and maintained in a humidified atmosphere at 37°C and 5% CO_2_. Later, the medium was removed and the culture was refreshed with new medium with 10% FCS. Cells were plated at a concentration of 3.5 x 10^5^ cells/mL and added with each compound (10 or 20 μM) for 24 or 72 h. Later, cells were harvested and lysed in an appropriate buffer containing 1% Triton X-100 and protease inhibitors. Proteins were quantitated by the BCA method (Pierce, Rockford, USA). Equal amounts of total cellular protein were resolved by SDS-polyacrylamide gel electrophoresis, with 10% acrylamide for PARP and 8% for Gli1 and SuFu. Blotted proteins were transferred by electroblotting to a PVDF membrane (Hoefer Pharmacia Biotech, San Francisco, USA) for 1 h at 100 v and 4°C. After a saturation step of 1 h with a solution of 5% nonfat dry milk and 0.1% TBST 10X in agitation at room temperature, anti-PARP, anti-β-actin, anti-Gli1 or anti-SuFu (Cell Signaling Technology, Boston, USA) antibodies were added to the PVDF membrane according to manufacturer’s instruction. On the day after, incubation with HRP-linked secondary antibodies was carried out for 1 h in agitation at room temperature and then HRP substrate was added (Bio-Rad Laboratories, Hercules, USA). Nonsaturated, immunoreactive bands were detected with a CCD camera gel documentation system (ChemiDocXRS, Bio-Rad Laboratories, Hercules, USA) and then quantitated with Image Lab ver.5.1 analysis software (Bio-Rad Laboratories, Hercules, USA). β-actin was used as loading control.

### Gene silencing

Gli1 gene silencing on K-562 cells was performed by inserting into cells a siRNA (AUAUCUUGCCCGAAGCAGGUAGUGC) towards Gli1 or a control scrambled siRNA owning the same CG ratio, at a final concentration of 30 nM by means of electroporation. Briefly, cells were centrifuged at 200g for 10 min. at room temperature, washed with sterile PBS and centrifuged again. Then, cells were resuspended in resuspension buffer R (Invitrogen, Carlsbad, USA) at a final density of 1 x 10^7^ cells/ml and siRNA towards Gli1 (or scrambled siRNA) was added at a final concentration of 30 nM. Then cells were electroporated using Neon^™^ Trasfection System (Invitrogen, Carlsbad, USA) and according to the following parameters: Pulse Voltage: 1350 v; Pulse Width: 10 ms; Pulse Number: 4; Cell Density: 3 x 10^7^. After electroporation, cells were plated on a 24-well plate at a concentration of 3 x 10^5^ cells/well in a final volume of 500 μL RPMI supplemented with 1% glutamine without antibiotics and with 10% FCS and incubated for 24 h in a humidified atmosphere at 37°C, 5% CO_2_ for further analysis.

### Statistical analysis

Reported data are Mean ± SEM of at least three independent experiment performed in triplicate. The statistical analysis was performed by Student’s t test using the Bonferoni correction for multiple test when appropriate. In all cases, only probability (p) values below 0.05 were considered significant.

## Supporting Information

S1 Appendix Synthesis of Smo antagonists. (PDF)

S1 Fig Synthesis of Smo antagonists. i. HCl, MeOH, r.t; ii. toluene, reflux; iii. NH_4_SCN, acetone, reflux.
(TIF)

## References

[1] Rowley JD. Letter: A new consistent chromosomal abnormality in chronic myelogenous leukaemia identified by quinacrine fluorescence and Giemsa staining. Nature 1973 6 1;243(5405):290–3. 4126434 10.1038/243290a0

[2] Shtivelman E, Lifshitz B, Gale RP, Canaani E. Fused transcript of abl and bcr genes in chronic myelogenous leukaemia. Nature 1985 6 13;315(6020):550–4. 2989692 10.1038/315550a0

[3] Ben-Neriah Y, Daley GQ, Mes-Masson AM, Witte ON, Baltimore D. The chronic myelogenous leukemia-specific P210 protein is the product of the bcr/abl hybrid gene. Science 1986 7 11;233(4760):212–4. 3460176 10.1126/science.3460176

[4] Daley GQ, Baltimore D. Transformation of an interleukin 3-dependent hematopoietic cell line by the chronic myelogenous leukemia-specific P210bcr/abl protein. Proc Natl Acad Sci U S A 1988 12;85(23):9312–6. 3143116 10.1073/pnas.85.23.9312 PMC282729

[5] Lambert GK, Duhme-Klair AK, Morgan T, Ramjee MK. The background, discovery and clinical development of BCR-ABL inhibitors. Drug Discov Today 2013 10;18(19–20):992–1000. 10.1016/j.drudis.2013.06.001 23769978

[6] Buchdunger E, Zimmermann J, Mett H, Meyer T, Muller M, Druker BJ, et al Inhibition of the Abl protein-tyrosine kinase in vitro and in vivo by a 2-phenylaminopyrimidine derivative. Cancer Res 1996 1 1;56(1):100–4. 8548747

[7] Druker BJ, Tamura S, Buchdunger E, Ohno S, Segal GM, Fanning S, et al Effects of a selective inhibitor of the Abl tyrosine kinase on the growth of Bcr-Abl positive cells. Nat Med 1996 5;2(5):561–6. 8616716 10.1038/nm0596-561

[8] FDA approves Gleevec for leukemia treatment. FDA Consum. 35 (6). 2001. 11692893

[9] Drugs Approved for Chronic Myelogenous Leukemia (CML). Available: http://www.cancer.gov/about-cancer/treatment/drugs/leukemia#7.

[10] Mahon FX, Deininger MW, Schultheis B, Chabrol J, Reiffers J, Goldman JM, et al Selection and characterization of BCR-ABL positive cell lines with differential sensitivity to the tyrosine kinase inhibitor STI571: diverse mechanisms of resistance. Blood 2000 8 1;96(3):1070–9. 10910924

[11] de LH, Apperley JF, Khorashad JS, Milojkovic D, Reid AG, Bua M, et al Imatinib for newly diagnosed patients with chronic myeloid leukemia: incidence of sustained responses in an intention-to-treat analysis. J Clin Oncol 2008 7 10;26(20):3358–63. 10.1200/JCO.2007.15.8154 18519952

[12] Jabbour E, Kantarjian HM, Abruzzo LV, O'Brien S, Garcia-Manero G, Verstovsek S, et al Chromosomal abnormalities in Philadelphia chromosome negative metaphases appearing during imatinib mesylate therapy in patients with newly diagnosed chronic myeloid leukemia in chronic phase. Blood 2007 10 15;110(8):2991–5. 17625066 10.1182/blood-2007-01-070045

[13] O'Hare T, Eide CA, Deininger MW. Bcr-Abl kinase domain mutations and the unsettled problem of Bcr-AblT315I: looking into the future of controlling drug resistance in chronic myeloid leukemia. Clin Lymphoma Myeloma 2007 3;7 Suppl 3:S120–S130. 17382021 10.3816/clm.2007.s.012

[14] Weisberg E, Manley PW, Breitenstein W, Bruggen J, Cowan-Jacob SW, Ray A, et al Characterization of AMN107, a selective inhibitor of native and mutant Bcr-Abl. Cancer Cell 2005 2;7(2):129–41. 15710326 10.1016/j.ccr.2005.01.007

[15] Jorgensen HG, Allan EK, Jordanides NE, Mountford JC, Holyoake TL. Nilotinib exerts equipotent antiproliferative effects to imatinib and does not induce apoptosis in CD34+ CML cells. Blood 2007 5 1;109(9):4016–9. 17213283 10.1182/blood-2006-11-057521

[16] Lombardo LJ, Lee FY, Chen P, Norris D, Barrish JC, Behnia K, et al Discovery of N-(2-chloro-6-methyl- phenyl)-2-(6-(4-(2-hydroxyethyl)- piperazin-1-yl)-2-methylpyrimidin-4- ylamino)thiazole-5-carboxamide (BMS-354825), a dual Src/Abl kinase inhibitor with potent antitumor activity in preclinical assays. J Med Chem 2004 12 30;47(27):6658–61. 15615512 10.1021/jm049486a

[17] Asaki T, Sugiyama Y, Hamamoto T, Higashioka M, Umehara M, Naito H, et al Design and synthesis of 3-substituted benzamide derivatives as Bcr-Abl kinase inhibitors. Bioorg Med Chem Lett 2006 3 1;16(5):1421–5. 16332440 10.1016/j.bmcl.2005.11.042

[18] O'Hare T, Shakespeare WC, Zhu X, Eide CA, Rivera VM, Wang F, et al AP24534, a pan-BCR-ABL inhibitor for chronic myeloid leukemia, potently inhibits the T315I mutant and overcomes mutation-based resistance. Cancer Cell 2009 11 6;16(5):401–12. 10.1016/j.ccr.2009.09.028 19878872 PMC2804470

[19] Hehlmann R. How I treat CML blast crisis. Blood 2012 7 26;120(4):737–47. 10.1182/blood-2012-03-380147 22653972

[20] Barnes DJ, Melo JV. Primitive, quiescent and difficult to kill: the role of non-proliferating stem cells in chronic myeloid leukemia. Cell Cycle 2006 12;5(24):2862–6. 17172863 10.4161/cc.5.24.3573

[21] Long B, Zhu H, Zhu C, Liu T, Meng W. Activation of the Hedgehog pathway in chronic myelogeneous leukemia patients. J Exp Clin Cancer Res 2011;30:8 10.1186/1756-9966-30-8 21235817 PMC3032744

[22] Hamilton A, Helgason GV, Schemionek M, Zhang B, Myssina S, Allan EK, et al Chronic myeloid leukemia stem cells are not dependent on Bcr-Abl kinase activity for their survival. Blood 2012 2 9;119(6):1501–10. 10.1182/blood-2010-12-326843 22184410 PMC3286213

[23] Crews LA, Jamieson CH. Selective elimination of leukemia stem cells: hitting a moving target. Cancer Lett 2013 9 10;338(1):15–22. 10.1016/j.canlet.2012.08.006 22906415

[24] Hurtz C, Hatzi K, Cerchietti L, Braig M, Park E, Kim YM, et al BCL6-mediated repression of p53 is critical for leukemia stem cell survival in chronic myeloid leukemia. J Exp Med 2011 10 24;208(11):2163–74. 10.1084/jem.20110304 21911423 PMC3201200

[25] Zhang H, Li H, Xi HS, Li S. HIF1alpha is required for survival maintenance of chronic myeloid leukemia stem cells. Blood 2012 3 15;119(11):2595–607. 10.1182/blood-2011-10-387381 22275380 PMC3311277

[26] Jagani Z, Dorsch M, Warmuth M. Hedgehog pathway activation in chronic myeloid leukemia. Cell Cycle 2010 9 1;9(17):3449–56. 20928937 10.4161/cc.9.17.12945

[27] Zhao C, Chen A, Jamieson CH, Fereshteh M, Abrahamsson A, Blum J, et al Hedgehog signalling is essential for maintenance of cancer stem cells in myeloid leukaemia. Nature 2009 4 9;458(7239):776–9. 10.1038/nature07737 19169242 PMC2946231

[28] Dierks C, Beigi R, Guo GR, Zirlik K, Stegert MR, Manley P, et al Expansion of Bcr-Abl-positive leukemic stem cells is dependent on Hedgehog pathway activation. Cancer Cell 2008 9 9;14(3):238–49. 10.1016/j.ccr.2008.08.003 18772113

[29] Merchant AA, Matsui W. Targeting Hedgehog—a cancer stem cell pathway. Clin Cancer Res 2010 6 15;16(12):3130–40. 10.1158/1078-0432.CCR-09-2846 20530699 PMC2888641

[30] Regl G, Kasper M, Schnidar H, Eichberger T, Neill GW, Philpott MP, et al Activation of the BCL2 promoter in response to Hedgehog/GLI signal transduction is predominantly mediated by GLI2. Cancer Res 2004 11 1;64(21):7724–31. 15520176 10.1158/0008-5472.CAN-04-1085

[31] Kenney AM, Rowitch DH. Sonic hedgehog promotes G(1) cyclin expression and sustained cell cycle progression in mammalian neuronal precursors. Mol Cell Biol 2000 12;20(23):9055–67. 11074003 10.1128/mcb.20.23.9055-9067.2000 PMC86558

[32] Kenney AM, Cole MD, Rowitch DH. Nmyc upregulation by sonic hedgehog signaling promotes proliferation in developing cerebellar granule neuron precursors. Development 2003 1;130(1):15–28. 12441288 10.1242/dev.00182

[33] Bigelow RL, Chari NS, Unden AB, Spurgers KB, Lee S, Roop DR, et al Transcriptional regulation of bcl-2 mediated by the sonic hedgehog signaling pathway through gli-1. J Biol Chem 2004 1 9;279(2):1197–205. 14555646 10.1074/jbc.M310589200

[34] Duman-Scheel M, Weng L, Xin S, Du W. Hedgehog regulates cell growth and proliferation by inducing Cyclin D and Cyclin E. Nature 2002 5 16;417(6886):299–304. 12015606 10.1038/417299a

[35] Lee J, Platt KA, Censullo P, Altaba A. Gli1 is a target of Sonic hedgehog that induces ventral neural tube development. Development 1997 7;124(13):2537–52. 9216996 10.1242/dev.124.13.2537

[36] Goodrich LV, Johnson RL, Milenkovic L, McMahon JA, Scott MP. Conservation of the hedgehog/patched signaling pathway from flies to mice: induction of a mouse patched gene by Hedgehog. Genes Dev 1996 2 1;10(3):301–12. 8595881 10.1101/gad.10.3.301

[37] Frank-Kamenetsky M, Zhang XM, Bottega S, Guicherit O, Wichterle H, Dudek H, et al Small-molecule modulators of Hedgehog signaling: identification and characterization of Smoothened agonists and antagonists. J Biol 2002 11 6;1(2):10 12437772 10.1186/1475-4924-1-10 PMC137065

[38] Tang T, Tang JY, Li D, Reich M, Callahan CA, Fu L, et al Targeting superficial or nodular Basal cell carcinoma with topically formulated small molecule inhibitor of smoothened. Clin Cancer Res 2011 5 15;17(10):3378–87. 10.1158/1078-0432.CCR-10-3370 21558397 PMC3113453

[39] Pellicano F, Simara P, Sinclair A, Helgason GV, Copland M, Grant S, et al The MEK inhibitor PD184352 enhances BMS-214662-induced apoptosis in CD34+ CML stem/progenitor cells. Leukemia 2011 7;25(7):1159–67. 10.1038/leu.2011.67 21483442 PMC3643208

[40] Samanta AK, Chakraborty SN, Wang Y, Schlette E, Reddy EP, Arlinghaus RB. Destabilization of Bcr-Abl/Jak2 Network by a Jak2/Abl Kinase Inhibitor ON044580 Overcomes Drug Resistance in Blast Crisis Chronic Myelogenous Leukemia (CML). Genes Cancer 2010 4;1(4):346–59. 10.1177/1947601910372232 20798787 PMC2927857

[41] Liao HF, Su YC, Zheng ZY, Jhih CC, Hou MH, Chao KS, et al Sonic hedgehog signaling regulates Bcr-Abl expression in human chronic myeloid leukemia cells. Biomed Pharmacother 2012 7;66(5):378–83. 10.1016/j.biopha.2011.12.008 22397755

[42] Ferretti E, De SE, Miele E, Laneve P, Po A, Pelloni M, et al Concerted microRNA control of Hedgehog signalling in cerebellar neuronal progenitor and tumour cells. EMBO J 2008 10 8;27(19):2616–27. 10.1038/emboj.2008.172 18756266 PMC2567402

[43] Babashah S, Sadeghizadeh M, Hajifathali A, Tavirani MR, Zomorod MS, Ghadiani M, et al Targeting of the signal transducer Smo links microRNA-326 to the oncogenic Hedgehog pathway in CD34+ CML stem/progenitor cells. Int J Cancer 2013 8 1;133(3):579–89. 10.1002/ijc.28043 23341351

[44] Manetti F, Faure H, Roudaut H, Gorojankina T, Traiffort E, Schoenfelder A, et al Virtual screening-based discovery and mechanistic characterization of the acylthiourea MRT-10 family as smoothened antagonists. Mol Pharmacol 2010 10;78(4):658–65. 10.1124/mol.110.065102 20664000

[45] Solinas A, Faure H, Roudaut H, Traiffort E, Schoenfelder A, Mann A, et al Acylthiourea, acylurea, and acylguanidine derivatives with potent hedgehog inhibiting activity. J Med Chem 2012 2 23;55(4):1559–71. 10.1021/jm2013369 22268551

[46] Roudaut H, Traiffort E, Gorojankina T, Vincent L, Faure H, Schoenfelder A, et al Identification and mechanism of action of the acylguanidine MRT-83, a novel potent Smoothened antagonist. Mol Pharmacol 2011 3;79(3):453–60. 10.1124/mol.110.069708 21177415

[47] Chen MH, Wilson CW, Li YJ, Law KK, Lu CS, Gacayan R, et al Cilium-independent regulation of Gli protein function by Sufu in Hedgehog signaling is evolutionarily conserved. Genes Dev 2009 8 15;23(16):1910–28. 10.1101/gad.1794109 19684112 PMC2725943

[48] Naldini A, Morena E, Pucci A, Miglietta D, Riboldi E, Sozzani S, et al Hypoxia affects dendritic cell survival: role of the hypoxia-inducible factor-1alpha and lipopolysaccharide. J Cell Physiol 2012 2;227(2):587–95. 10.1002/jcp.22761 21448921

[49] Lee J, Giordano S, Zhang J. Autophagy, mitochondria and oxidative stress: cross-talk and redox signalling. Biochem J 2012 1 15;441(2):523–40. 10.1042/BJ20111451 22187934 PMC3258656

[50] Warzecha J, Bonke L, Koehl U, Munkelt D, Gottig S, Percic D, et al The hedgehog inhibitor cyclopamine induces apoptosis in leukemic cells in vitro. Leuk Lymphoma 2008 12;49(12):2383–6. 10.1080/10428190802510315 19052992

[51] Katagiri S, Tauchi T, Okabe S, Minami Y, Kimura S, Maekawa T, et al Combination of ponatinib with Hedgehog antagonist vismodegib for therapy-resistant BCR-ABL1-positive leukemia. Clin Cancer Res 2013 3 15;19(6):1422–32. 10.1158/1078-0432.CCR-12-1777 23319824

[52] Untergasser A, Cutcutache I, Koressaar T, Ye J, Faircloth BC, Remm M, et al Primer3—new capabilities and interfaces. Nucleic Acids Res 2012 8;40(15):e115 22730293 10.1093/nar/gks596 PMC3424584

[53] Koressaar T, Remm M. Enhancements and modifications of primer design program Primer3. Bioinformatics 2007 5 15;23(10):1289–91. 17379693 10.1093/bioinformatics/btm091

