# Supplementary material for: Novel Acylguanidine Derivatives Targeting Smoothened Induce Antiproliferative and Pro-Apoptotic Effects in Chronic Myeloid Leukemia Cells
Source: PLoS One. 2016 Mar 2;11(3):e0149919. doi: 10.1371/journal.pone.0149919 (PMC4774938; doi:10.1371/journal.pone.0149919)
Supplement: S1 Appendix — (PDF) [file pone.0149919.s001.pdf]

S1 Appendix

*Synthesis of Smo antagonists*. Five compound (namely MRT83, MRT92, MRT94, MRTX, and MRTY) have been synthesized following previously developed protocols.[1S,2S] In detail, the guanidine derivatives MRT83, MRT92, MRT94 and MRTX have been obtained reacting cyanamide 1 with the proper aniline 2 as chlorohydrate in boiling toluene (S1 figure). All the guanidines have been converted into their chlorohydrate salts by treatment with HCl in MeOH. Thiourea MRTY has been prepared refluxing aniline 2 and acyl chloride 3 in the presence of $NH_4SCN$.

*Synthesis of acylguanidine derivatives MRTX, MRT83, MRT92, MRT94.* Cyanamide 1 (210 mg, 0.9 mmol) was suspended in toluene (40 mL) and treated with the proper aniline chlorohydrate (0.99 mmol) at reflux for 3 h. The reaction mixture was cooled down to r.t. and the precipitate obtained was filtered over B· ner washing with $Et_2O$ (2 x 20 mL). The solid obtained was directly treated with 2M HCl in MeOH overnight. After evaporation, expected products were obtained as pale yiellow solids.

*N*-(*N*-(3-Benzamido-4-methylphenyl)carbamimidoyl)-3,4,5-trimethoxybenzamide chlorohydrate (MRTX). Yield: 51%. M.p. 138–C (as HCl). $^{1}$H NMR (300 MHz, DMSO, d6) d 12.41 (m, 1 H), 11.54 (m, 1H), 10.05 (s, 1H), 9.62 ( brs, 1H), 8.87 (m, 1H), 8.01 (m, 2H), 7.58-7.53 (m, 10H), 3.91 (s, 6H), 3.79 (s, 3H). $^{13}$C NMR (75 MHz, DMSO, d6) $\delta$ = 165.8, 152.8, 141.3, 136.9, 134.3, 132.2, 121.9, 128.9. 127.7, 127.1, 121.2, 119.1, 106.1, 60.9, 56.2, 17.3. MS-ESI (m/z): 463 [M - Cl]$^+$.

*N*-(2-Methyl-5-(3-(3,4,5-trimethoxybenzoyl)guanidino)phenyl)biphenyl-4-carboxamide hydrochloride (MRT83). Yield: 58%. M.p. 155-159 (as HCl). $^{1}$H NMR (400 MHz, DMSO, d6) $\delta$ = 9.18 (bs, 1 H), 9.00 (bs, 1H), 8.06 (bs, 2H), 7.80-7.10 (m, 14H), 3.70 (s, 6H), 3.68 (s, 3H), 2.77 (s, 3 H). $^{13}$C NMR (100 MHz, DMSO, d6) $\delta$ 176.0, 168.2, 165.0, 160.0, 156.5, 150.0, 146.3, 139.0, 137.4, 133.1, 131.8, 131.4, 131.0, 128.9, 128.6, 127.1, 127.0, 126.4, 123.5, 121.0, 119.3, 118.5, 116.0, 61.3, 56.0, 34.9. MS-ESI (m/z): 539 [M - Cl]$^+$

*(E)*-3,4,5-trimethoxy-*N*-(*N*-(4-methyl-3-(4-styrylbenzamido)phenyl)carbamimidoyl) benzamide (MRT94). Yield 73% M.p. 221-223 °C (as HCl). $^{1}$H NMR ($CDCl_3$, 400 MHz) δ 12.33 (bs, 1H), 9.97 (bs, 1H), 8.44 (bs, 1H), 7.93 (m, 3H), 7.63 (s, 2H), 7.59 (d, J 8.2 Hz, 2H), 7.54 (d, J 8.2 Hz, 2H), 7.39 (bt, J 7.4 Hz, 2H), 7.31 (m, 2H), 7.20 (d, J 16.2 Hz, 1H), 7.11 (d, J 16.2 Hz, 1H), 7.07 (dd, J 8.2, 1.9 Hz, 1H), 3.99 (s, 6H), 3.95 (s, 3H), 2.32 (s, 3H). $^{13}$C NMR ($CDCl_3$, 100 MHz) δ 168,7, 165.7, 155.1, 153.5, 143.7, 141.6, 137.5, 136,8, 132.7, 132.4, 131.5, 131.0, 130.1, 129.0, 128.5, 127.9, 127.3, 127.0, 126.9, 125.0, 122.1, 120.4, 106.7, 61.1, 57.1, 17.8. LRMS-ESI (m/z) $[M-Cl]^+$ calcd: 565.2, found: 565.2..

3,4,5-trimethoxy-*N*-(*N*-(4-methyl-3-(4-phenethylbenzamido)phenyl)carbamimidoyl) benzamide hydrochloride (MRT92). Yield 70% M.p 186-189 °C (as HCl). $^{1}$H NMR ($CDCl_3$, 400 MHz) δ 12.64 (bs, 1H), 11.93 (bs, 1H), 10.01 (bs, 1H), 8.30 (bs, 1H), 7,90 (m, 3H), 7.64 (bs, 2H), 7.37-6.98 (m, 9H), 4.00 (s, 6H), 3.95 (s, 3H), 2.97 (m, 4H), 2.31 (s, 3H). $^{13}$C NMR ($CDCl_3$, 100 MHz) δ 168.6, 166.0, 155.1, 153.5, 146.9, 143.7, 141.1, 137.5, 132.7, 131.6, 131.0, 130.1, 129.2, 128.6, 127.6, 124.9, 124.9, 122.1, 120.3, 106.8, 61.1, 57.1, 37.8, 37.5, 17.8; LRMS-ESI (m/z) $[M-Cl]^+$ calcd: 567.3; found: 567.2.

Synthesis of *N*-(2-methyl-5-(3-(3,4,5-trimethoxybenzoyl)thioureido)phenyl)biphenyl-3-carboxamide (MRTY). Ammonium thiocyanate (125 mg, 1.63 mmol) was dissolved in dry acetone (4 mL). To this stirred solution, 3,5-dimethoxybenzoyl chloride 3 (320 mg, 1.36 mmol) was added, refluxing the reaction mixture for 20 min. After the solution became clear, benzamide 2d (275 mg, 1.30 mmol) was added, and the reaction mixture refluxed for 60 min. Monitoring of the reaction was carried out by TLC, using petroleum ether:AcOEt=1:4. Upon disappearance of the starting amine, the reaction mixture was poured into a beaker containing ice. The resulting precipitate was filtered and crystallized from methanol, affording MRTY in 69% yield. M.p. 193-194 °C. $^{1}$H NMR (300 MHz, $CDCl_3$) δ = 12.56 (brs, 1H), 9.01 (brs, 1H), 8.38 (brs, 1H), 8.13 (brs, 1H), 7.84-7.09 (m, 15H), 3.95 (s, 9H), 2.37 (s,

3H). 13C NMR (75 MHz, CDCl3) δ = 178.3, 167.0, 165.4, 161.3, 156.1, 153.8, 143.1, 136.5, 131.1, 131.0, 130.6, 130.6, 130.4, 129.5, 129.2, 128.8, 127.0, 124.8, 121.5, 120.3, 119.5, 118.2, 105.4, 61.4, 56.8, 18.0. MS-ESI (m/z): 556 $[M+H]^+$

1S) Solinas, A.; Faure, H.; Roudaut, H.; Traiffort, E.; Schoenfelder, A.; Mann, A.; Manetti, F.; Taddei, M.; Ruat, M. Acylthiourea, acylurea, and acylguanidine derivatives with potent hedgehog inhibiting activity. *J. Med. Chem.* 2012, *55*, 1559-1571.

2S) Hoch, L.; Faure, H.; Roudaut, H.; Schoenfelder, A.; Mann, A.; Girard, N., Bihannic, L.,; Ayrault, O.; Petricci, E.; Taddei, M.; Rognan, D.; Ruat, M. *FASEB J.* 2015, *29*, 1817-1829.
