# Supplementary figures and images for: Novel Acylguanidine Derivatives Targeting Smoothened Induce Antiproliferative and Pro-Apoptotic Effects in Chronic Myeloid Leukemia Cells
Source: PLoS One. 2016 Mar 2;11(3):e0149919. doi: 10.1371/journal.pone.0149919 (PMC4774938; doi:10.1371/journal.pone.0149919)

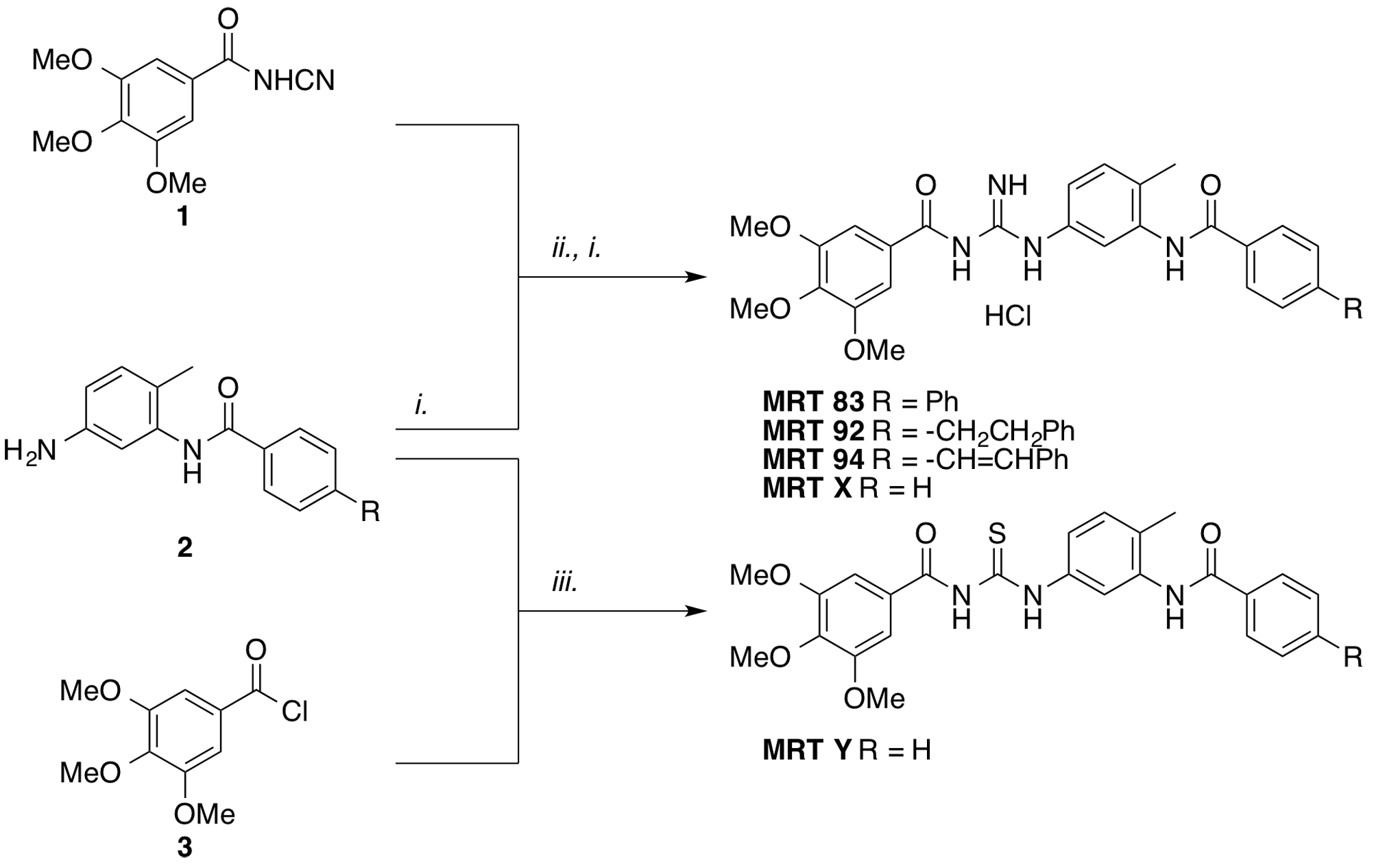

Supplement: S1 Fig — i. HCl, MeOH, r.t; ii. toluene, reflux; iii. NH4SCN, acetone, reflux. (TIF) [file pone.0149919.s002.tif]
